# Cu_2_O photocathodes with band-tail states assisted hole transport for standalone solar water splitting

**DOI:** 10.1038/s41467-019-13987-5

**Published:** 2020-01-16

**Authors:** Linfeng Pan, Yuhang Liu, Liang Yao, Dan Ren, Kevin Sivula, Michael Grätzel, Anders Hagfeldt

**Affiliations:** 1 Laboratory of Photomolecular Science, Institute of Chemical Sciences and Engineering, École Polytechnique Fédérale de Lausanne (EPFL), Lausanne, CH-1015 Switzerland; 2 Laboratory of Photonics and Interfaces, Institute of Chemical Sciences and Engineering, École Polytechnique Fédérale de Lausanne (EPFL), Lausanne, CH-1015 Switzerland; 3 Laboratory of Molecular Engineering of Optoelectronic Nanomaterials, Institute of Chemical Sciences and Engineering, École Polytechnique Fédérale de Lausanne (EPFL), Lausanne, CH-1015 Switzerland

**Keywords:** Artificial photosynthesis, Solar fuels, Photocatalysis

## Abstract

Photoelectrochemical water splitting provides a promising solution for harvesting and storing solar energy. As the best-performing oxide photocathode, the Cu_2_O photocathode holds the performance rivaling that of many photovoltaic semiconductor-based photocathodes through continuous research and development. However, the state-of-the-art Cu_2_O photocathode employs gold as the back contact which can lead to considerable electron-hole recombination. Here, we present a Cu_2_O photocathode with overall improved performance, enabled by using solution-processed CuSCN as hole transport material. Two types of CuSCN with different structures are synthesized and carefully compared. Furthermore, detailed characterizations reveal that hole transport between Cu_2_O and CuSCN is assisted by band-tail states. Owing to the multiple advantages of applying CuSCN as the hole transport layer, a standalone solar water splitting tandem cell is built, delivering a solar-to-hydrogen efficiency of 4.55%. Finally, approaches towards more efficient dual-absorber tandems are discussed.

## Abstract (web summary)

While solar-to-fuel conversion offers a promising technology to produce energy, device components can limit light absorption and reduce performances. Here, authors show copper thiocyanate to assist hole transport in photoelectrodes and enable a 4.55% solar-to-hydrogen efficiency in tandem devices.

## Introduction

As one of the solutions for future energy demand, solar energy shows promise to substitute fossil fuels without sacrificing our standard of living. Photoelectrochemical (PEC) solar energy conversion further addresses the intermittency of sunlight to meet the need for long-term energy storage. In the most studied field of solar-to-hydrogen (STH) conversion, oxide-based photoelectrodes are attractive due to their high abundance, economical fabrication, and excellent stability^1–3^. Among all oxide photocathodes where hydrogen is generated during solar water splitting, Cu_2_O photocathodes hold the champion performance rivaling that of many photovoltaic (PV) semiconductor-based photocathodes^4^. However, Rome was not built in a day. Starting from the ground-breaking work by Paracchino et al. where the Cu_2_O photocathode was protected by atomic layer deposition (ALD) layers of TiO_2_, Cu_2_O photocathodes are giving meaningful photocurrent with hours of stable operation^5^. Subsequently, efforts were made on surface catalysts to enable better stability in electrolyte of various pH values^6–8^. To achieve optimized light absorption and minority carrier transfer, the photoabsorber Cu_2_O was nanostructured via high-temperature processes, resulting in simultaneously enhanced photocurrent density and stability^9,10^. Most recently, another significant advance was realized by building the more efficient coaxial heterojunction of Ga_2_O_3_/Cu_2_O^11,12^. With excellent photovoltage of around 1 V versus reversible hydrogen electrode (RHE), which is the highest among all single-junction photocathodes, an all-oxide standalone tandem system with 3% STH efficiency was demonstrated, proving the potential of Cu_2_O photocathodes for solar energy conversion commercialization^12^.

Until now, state-of-the-art Cu_2_O photocathodes still employ gold as the back contact due to the matching between gold work function and Cu_2_O valence band energy level^5,10,12^. In addition to its scarcity, gold is not a hole-selective contact, which will cause considerable amount of recombination. Moreover, Au absorbs and refelect strongly from visible to infrared light, leading to the difficulty of making transparent Cu_2_O photocathodes^13^. Since Cu_2_O is often used as front absorber due to its relatively large bandgap, the opacity would hinder the establishment of an efficient tandem based on Cu_2_O photocathodes. To our best knowledge, only nickel oxide-based materials have been studied as the hole-transport layer (HTL) for the Cu_2_O photocathode. Nevertheless, a two-step process, including thermal annealing, was required in both cases.

In this study, we report the use of CuSCN as an effective hole-transport material for Cu_2_O photocathodes. Overall PEC performance improvement was achieved with solution-processed CuSCN layers, featuring excellent fill factors. Material, optical and electronic properties of two types of CuSCN with different structures were characterized and compared. Furthermore, band-tail assisted hole-transport was uncovered for efficient hole-selective conduction between Cu_2_O and CuSCN. To show the multiple advantages of employing CuSCN as the HTL, a PEC-PV tandem was optimized and demonstrated, delivering 4.55% STH efficiency.

## Results

### Characterization of CuSCN films

**Fig. 1:**
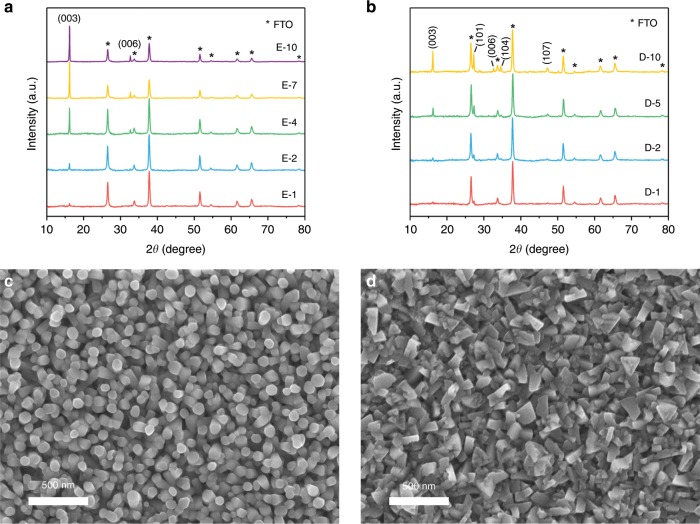
XRD patterns and SEM imaging of CuSCN films. **a** XRD patterns of CuSCN-E on FTO substrates with various electrodeposition duration. **b** XRD patterns of CuSCN-D on FTO substrates with various electrodeposition duration. Numbers in sample names denote the electrodeposition duration in minutes. Indexes were extracted from PDF cards of #01-077-0451 for FTO and #29-0581 for CuSCN. **c** Top-view image of CuSCN-E with 2 min electrodeposition. **d** Top-view image of CuSCN-D with 2 min electrodeposition. Scale bar: 500 nm.

CuSCN has extensive applications in opto-electronic devices^14^. Apart from its exceptional optical transparency, chemical stability, and hole-transport properties, processing versatility is also considered one of the merits of CuSCN for manufacturing electronic devices. Electrodeposition was selected as the method because of its low cost and high compatability with large-scale production. Moreover, the solution-based nature allows for precise control of morphology, thickness and composition of the deposited film^15^. Previous reports on electrodeposition of CuSCN on a rotating disk electrode revealed that the growth of nanostructured CuSCN is limited by mass transport^16,17^. Extensive varied deposition parameters have been studied including bath composition, temperature, pH values to tune its morphology, orientation, and nanostructures^16–21^. In order to attain precise stoichiometry and reliable successful deposition rate, we maintained the 1:1 molar ratio of [Cu^2+^]:[SCN^-^] in both electrolytes but differed the chelating agent, which will effectively change the solution pH^18,20^. Indeed, a solution containing ethylenediaminetetraacetic acid (EDTA) showed a pH value of 1.6, whereas one with diethanolamine (DEA) gave a pH value of 8.2. Phase composition and crystalline structure of the resulted films (CuSCN-E and CuSCN-D for EDTA and DEA, respectively) were analyzed by X-ray diffractometry (XRD) as shown in Fig. 1a, b. Both films show diffraction peaks that are indexed to the rhombohedral β-CuSCN (JCPDS No. 29-0581). No impurity peaks are visible other than those of fluorine-doped tin oxide (FTO) substrates (marked with asterisks, JCPDS No. 01-077-0451). While the CuSCN-D shows four major diffraction peaks of (003), (101), (104) and (107) featuring (101) and (003) peaks, CuSCN-E has a preferred orientation along (001) direction with sharp shapes indicating high crystallinity. The samples prepared in acidic bath show only (001) direction peaks of increasing intensity with longer deposition. In contrast, CuSCN-D shows both pronounced peaks of (003) and (101) as deposition time increases. Comparing the two films with 10 min electrodeposition, relative intense peaks are obtained with CuSCN-E series, implying better crystallinity. These results associate properly with scanning electron microscope (SEM) images.

Both top-view and cross-section SEM images are collected on samples with various deposition duration. Similar to electrodeposition in other acidic electrolyte, samples produced with EDTA show dense and vertical nanorods morphology (Fig. 1c)^19,20,22^. As expected in XRD analysis, the films exhibit well-defined columnar crystals with uniform diameters. The strong preferential orientation along *c*-axis is logically corresponding to the longitudinal direction of the nanorods^19^. While the deposition duration gets longer, nanorod diameters increase from ~100–300 nm, losing its diameter uniformity (Supplementary Fig. 1). This shows that the nanorods are formed due to mass transport limitation, since the reactant, [Cu(SCN)^+^], is quickly depleted among the nanorods so a pillar-like structure is developed^16^. Corresponding cross-section images (Supplementary Fig. 2) show not only the thickening of the film but also the enhanced density, as the space among the columns are filled gradually. Figure 1d shows an image of CuSCN-D. Rugged grains are closely packed with well-defined structure. As the films get thicker, grains get larger with more noticeable plain surface and broken edges (Supplementary Fig. 3). The drastic structure difference exhibited here has been explained by the difference between the formation of different copper complex depending on pH^20,23^. A closer look at the cross-section images (Supplementary Fig. 4) reveals that though the surface of film is rough, the film is continuous, which promises the reduction of leakage current when functioning as the HTL. Thickness growth rate was estimated using linear fitting of SEM cross-section thicknesses from three independent batches prepared with the same configuration. Growth rates of 141.5 nm min^−1^ and 150.0 nm min^−1^ were, respectively, estimated for EDTA and DEA assisted depositions (Supplementary Fig. 5).

A thorough X-ray photoelectron spectroscopy (XPS) study has been carried out on the CuSCN films. The survey results resemble the ones in literature validating the presence of Cu, S, C, and N, and indicate a trace amount of oxygen, which is due to inevitable contamination on the surface of the samples, Supplementary Fig. 6^24–26^. High-resolution spectra of selected elements in CuSCN films prepared with different chelating agents were collected and presented in Supplementary Figs. 7 and 8. It is conceivable that covalent carbon binding in CuSCN will substantially contribute to the C 1s spectra, therefore the peak at 284.8 eV, which is ascribed to aliphatic carbon contamination, was used for calibration after deconvolution. The other major peak in C 1s spectra located at 286 eV corresponds to the sp-state carbon in –C≡N, while the small one at 288.2 is due to C–O bond of impurities (Supplementary Figs. 7a and 8a)^24,27^. The peak in the S 2p region is formed by doublets of S 2p_3/2_ and S 2p_1/2_, which were deconvoluted to two peaks at 163.5 eV and 164.6 eV. Both of them can be ascribed to sulfur in –S-C form (Supplementary Figs. 7b and 8b). Similar patterns are also acquired from both CuSCN films for N 1s spectra where the main deconvoluted peak at 398.3 eV probably corresponds to nitrogen in nitrile form (Supplementary Figs. 7c and 8c). In both Cu 2p_3/2_ spectra, the curve peaks at 932.8 eV, which can be assigned to Cu^1+^ in CuSCN (Supplementary Figs. 7d and 8d). No obvious distinction occurs between these two films until we focus on the binding energy of 934.7 eV, where only the CuSCN-D show a relatively large peak. This peak has been observed and usually is interpreted as either CuO, Cu_2_O, or Cu^2+^^24,26–28^. We then carefully checked the O 1s spectra of both samples as shown in Supplementary Fig. 9. The prominent peaks at 531.6 eV is inevitable on samples exposed to the atmosphere due to contamination such as water. Peaks at binding energies of 529.6 eV and 530.5 eV, which, respectively, represent O 1s core level features of CuO and Cu_2_O, did not appear^28^. However, an intriguing peak at 533.4 eV emerges only in the O 1s spectrum of CuSCN-D. This peak is regarded as evidence of metal carbonates, metal hydroxides, or organic C–O bond^29,30^. Combining with the exclusive Cu signals of the CuSCN-D, we suggest the presence of Cu^2+^ carbonate hydroxide, which is probably derived from Cu^2+^ species of CuSCN-D in the alkaline electrolyte. This result correlates with the hysteresis we observed in CuSCN-D during the Space-Charge Limited Current (SCLC) measurement discussed in the following sections. In addition to the XRD analysis, it implies that CuSCN-D features a relatively defective crystal with unconsolidated structure compared to the highly crystalline columnar CuSCN-E.

### Photocathode performance

**Fig. 2:**
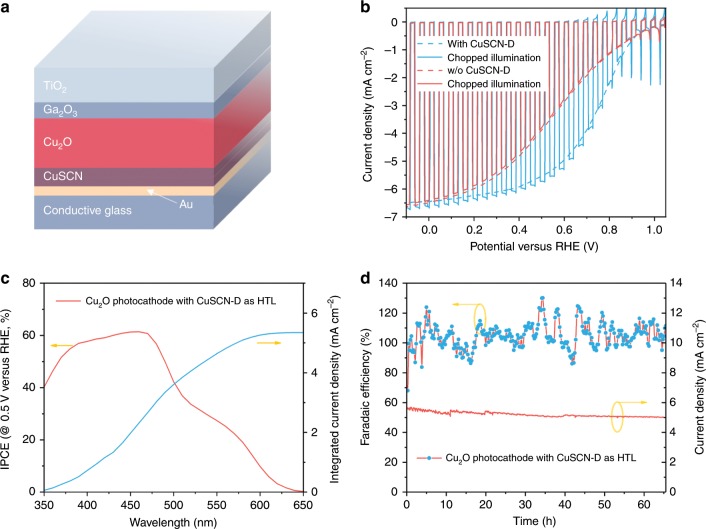
Structure and PEC performance of CuSCN-incorporated Cu_2_O photocathodes. **a** A schematic diagram of the CuSCN-incorporated Cu_2_O photocathode configuration. **b** Current density-potential (*J-E)* responses of Cu_2_O photocathodes with or w/o CuSCN-D layer under simulated one-sun illumination (air mass 1.5 G spectrum) in pH 5 buffered electrolyte (scan rate 10 mV/s). **c** Wavelength-dependent IPCE and integrated current density of Cu_2_O photocathodes with CuSCN-D layer. All photocathode tests are carried out on photocathodes with 2 min electrodeposited CuSCN-D layers. **d** Stability test at fixed bias of 0.5 V versus RHE and corresponding Faradaic efficiency for hydrogen evolution in pH 5 buffered electrolyte under.

Cu_2_O, Ga_2_O_3_, and TiO_2_ layers were deposited onto CuSCN in sequence to construct the photoelectrode with structure shown in Fig. 2a^12^. The heterojunction was built by marrying p-type Cu_2_O and n-type Ga_2_O_3_, buried by the surface TiO_2_ protection layer. RuO_x_ was photoelectrochemically deposited as the hydrogen evolution catalyst^8,10^. The new photocathode was tested with the conventional one (without CuSCN layer) by linear-sweep voltammetry (LSV) in pH 5 buffer solution. The LSV curves are shown in Fig. 2b. Distinct from the performance of conventional samples, which exhibits onset potential of 1 V versus RHE and current density of around 6.4 mA cm^−2^ at 0 V versus RHE with relatively poor fill factor, the photocathode with CuSCN layer shows globally enhanced photocurrent featuring excellent fill factor. Specifically, the current density of the new photocathode at 0.6 V versus RHE is around 5.3 mA cm^−2^ comparing to 3 mA cm^−2^ of the standard one. The PEC performance tested under chopped illumination (solid line) confirmed that both photocathodes are giving photocurrent instead of corrosion current. In the following systematic study, we varied the film thicknesses of both films by altering electrodeposition duration and tested their PEC behavior (Supplementary Fig. 13). Performance of Cu_2_O photocathodes on bare FTO was also recorded and discussed in supplementary information (Supplementary Figs. 10, 11, and 12). Though both types of CuSCN films show best performance with a 2 min deposition time, the Cu_2_O photocathodes with CuSCN-D shows drastic photocurrent difference when changing deposition duration. We believe this phenomenon has strong connection to the structure of the films. Since CuSCN-E possesses nanorod morphology, the minimum deposition time required for complete coverage may be significantly higher than that for CuSCN-D. Additionally, the growth rate of CuSCN-D is larger than that of CuSCN-E and the orientation could contribute to the variation as well. Nevertheless, the best cases with both films show improved fill factor, highlighting the 2-min CuSCN-D with overall enhanced PEC performance. The early onset potential with enhanced fill factor could boost the photocathode performance significantly in applications such as tandem devices and photo-redox reactions.

Figure 2c presents the wavelength-dependent monochronomatic incident photon-to-current conversion efficiency (IPCE), revealing the photocathode spectral response. Note that the IPCE was recorded at 0.5 V versus RHE as this value is more meaningful for tandem configurations. IPCE values were observed rising from 650 nm to around 60% at 470 nm, where the lowest direct allowed optical gap lies. This demonstrates the outstanding quantum efficiency of Cu_2_O photocathodes^31,32^. Importantly, the integrated IPCE values with the air mass (AM) 1.5 G spectrum yields current density of around 5.4 mA cm^−2^, validating the absence of any significant spectral mismatch between our light source and sunlight. Finally, the whole device was subjected to long-term operation with gas quantification, Fig. 2d. After 60 h of continuous test, the photocurrent drops from 5.3 mA cm^−2^ to ~5.0 mA cm^−2^. Despite the large efficiency fluctuation caused by irregular bubble release and small cell headspace, the average Faradaic efficiency remained at around 100% during the whole test, showing that all the observed currents can be attributed to the generation of hydrogen.

### Hole conducting mechanism

To understand the PEC behavior, both films are characterized by optical, electrochemical, and electronic measurement. Examination of transmittance are carried out on CuSCN coated FTO glass. As shown in Supplementary Fig. 14, CuSCN films prepared with short deposition duration (1 min and 2 min) show excellent transparency for wavelengths longer than 400 nm regardless of the choices of the chelating agents. While films get thicker with longer deposition, transparency decreases. To clearly see the wavelength-dependent absorption, absorbance spectra were obtained by converting the transmittance data, Supplementary Fig. 15. A steep absorption line arises at around 360 nm, which separates the UV region and visible region and their distinct absorbing behaviors. However, for thicker films, absorption extends into the visible part. Absorption data were then used to extract optical bandgap by plotting (αhν)^n^ against hν according to the Tauc Equation,

1 $$\begin{array}{*{20}{c}} {\alpha {\mathrm{h}}\nu = {\mathrm{A}}\left( {{\mathrm{h}}\nu - E_G} \right)^{1/n}} \end{array}$$

where “A” is proportionality constant; “hν” is photon energy displayed in “eV” and “E_g_” refers to the bandgap of the material. Though the question on the nature of the CuSCN bandgap remains open, we consider it direct (*n* = 2) as suggested in most cases^24,33^. The linear region is extrapolated to the *x*-axis. The enlarged fitting with thinner films is shown in the inset. The optical bandgaps for all CuSCN films is 3.84–3.95 eV, which agrees with reported results for p-type CuSCN (Supplementary Fig. 16)^19,24,33^. A bandgap of 2.02 eV was found for Cu_2_O with the above method (Supplementary Fig. 17). The reflectance data were also collected showing negligible reflection from the CuSCN films (Supplementary Fig. 18).

**Fig. 3:**
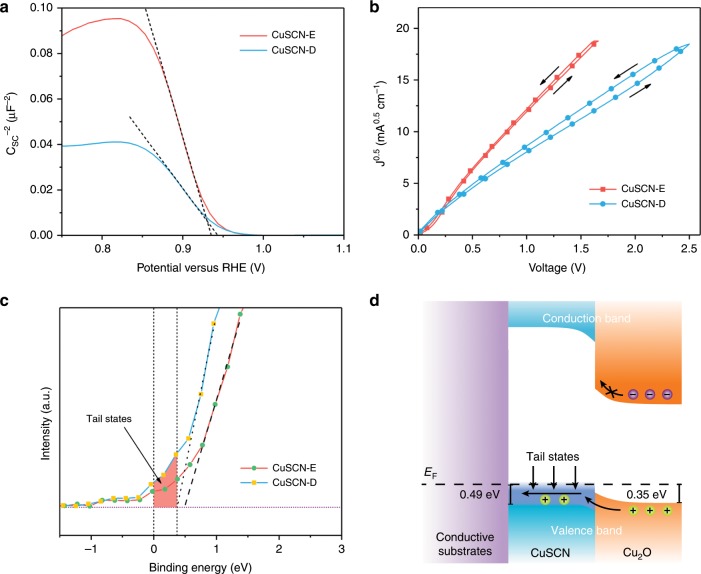
Electronic properties of CuSCN layers and hole-transport mechanism. **a** Mott-Schottky plot of CuSCN layers tested in 1 M Na_2_SO_4_ solution. **b** *J*^*0.5*^-*V* plots for CuSCN-based hole only devices. **c** Enlarged valence band spectra with shadowed area denoting the band-tail states. **d** Band energy diagrams showing the band-tail assisted hole transport between Cu_2_O and CuSCN.

To evaluate the electric conductivity of the films, we acquired the majority carrier density and mobility by Mott-Schottky and SCLC methods, respectively. According to the Mott-Schottky equation,

2 $$\begin{array}{*{20}{c}} {\frac{1}{{C^2}} = \frac{2}{{\varepsilon \varepsilon _0eN_AA^2}}\left( { - V + V_{fb} - \frac{{kT}}{e}} \right)} \end{array}$$

where *C* is the interfacial capacitance, *ε* is the dielectric constant of CuSCN, *ε*_0_ is the vacuum permittivity, *N*_*A*_ is the density states of acceptors in CuSCN, *V* is the applied potential, and *V*_*fb*_ is the flat band potential. The negative slopes of the linear part are clearly indicating the p-type nature of CuSCN films (Fig. 3a). By calculating the slopes and assuming the dielectric constant is invariable, one can obtain the hole concentrations for both films. However, a flat surface does not reflect the real surface morphology, which may lead to an overestimation of the carrier density^19^. Therefore, we assessed surface roughness by measuring the double-layer capacitance in quiescent electrolyte and compared it to that of ideally smooth CuSCN surface whose roughness is assumed to be unity. Cyclic voltammograms were collected with various scan rates in a potential window where the Faradaic current is minimal (Supplementary Fig. 19). The charging current, *i*_*c*_, is used to estimate the double-layer capacitance, *C*_*dl*_, using the following equation:

3 $$\begin{array}{*{20}{c}} {i_c = \nu C_{dl}} \end{array}$$

With the cylindrical CuSCN structure, the specific capacitance of CuSCN-E is around seven times larger than that of CuSCN-D (Supplementary Fig. 20). Assuming a specific capacitance of 20 μF cm^−2^, the roughness factors are calculated to be 21.29 and 4.165 for CuSCN-E and CuSCN-D, respectively^34^. With the correction of surface roughness, if ε (ε = 5.1) is considered constant, the carrier density of CuSCN-E and CuSCN-D are correspondingly determined to be 8.65 × 10^16^ cm^−3^ and 1.59 × 10^18^ cm^−3^^35^. The hole mobility was obtained from the trap-free SCLC regime of hole only devices (FTO/Au/CuSCN-E or CuSCN-D/MoO_3_/Ag, see details in the experimental section). The current density-voltage (*J*–*V*) characteristics of the hole only devices are exhibited in Fig. 3b. In the trap-free SCLC regime, the current density is known to follow the Mott-Gurney Law as follows,

4 $$\begin{array}{*{20}{c}} {J = \frac{8}{9}\mu _{\mathrm{h}}\varepsilon \varepsilon _0\frac{{V^2}}{{L^3}}} \end{array}$$

where *μ*_h_ is free carrier mobility, and *L* is the CuSCN film thickness. *ε*_0_ and *ε* is the vacuum permittivity and dielectric constant (reported to be 5.1 for CuSCN^35^,) respectively. By fitting the *J*–*V* curves, the *μ*_h_ of CuSCN-E and CuSCN-D is calculated to be 0.78 ± 0.06 and 0.34 ± 0.06 cm^2^ V^−1^ s^−1^, respectively. Both of these values are among the best of p-type semiconductors^36,37^. The excellent hole mobilities of CuSCN-E and CuSCN-D are favorable for extracting photo-generated holes and suppressing the interfacial recombination. It is noticeable that CuSCN-D based hole only devices show a more pronounced hysteresis phenomenon, compared to the devices based on CuSCN-E. Such a hysteresis phenomenon has often been observed in ion-contained devices, such as the emerging perovskite solar cells^38^. As previous discussions evidenced that CuSCN-E has higher crystallinity while CuSCN-D shows relatively loose structure bearing more defects that support the predominant p-type conductivity, the more pronounced hysteresis curves in CuSCN-D hole only devices is interpreted in terms of the migration of residual ions considering also that a thick CuSCN layer was used for hole only devices^14^. The hole conductivity (*σ*_h_) of CuSCN-E and CuSCN-D is estimated from the equation,

5 $$\begin{array}{*{20}{c}} {\sigma = eN_A\mu _h} \end{array}$$

where *e* is the elementary charge, *N*_*A*_ is the density states of acceptors in CuSCN. *σ*_*h*_ is increases 8 times from 0.011 S cm^−1^ of CuSCN-E films to 0.087 S cm^−1^ of CuSCN-D counterparts, which is similar to the reported results and presumably led to the better fill factor observed during PEC testing ^39^.

The XPS valence band spectra were acquired to analyze the electronic structures of CuSCN. The characteristic valence band signals show excellent consistency with the reported density of states calculations concerning number of featured peaks and energy positions after subtracting a Shirley background^40–43^. The prominent peak of Cu 3d orbital is well-resolved at around 3eV whereas other features arising between 3 eV and 12 eV can be attributed to the covalent bonding in the CuSCN (Cu-S, S-C, and C≡N bonds), Supplementary Fig. 21. The VBMs are found to be 0.49 eV for CuSCN-E and 0.37 eV for CuSCN-D relative to the Fermi level by linear extrapolation, which corresponds well with reported results acquired from both XPS and ultraviolet photoelectron spectroscopy (UPS), Fig. 3c^41,42,44^. (1.2) Small barriers will be formed between Cu_2_O/CuSCN interface during hole transport, as the valence band maximum (VBM) of Cu_2_O is ~0.35 eV, Supplementary Fig. 22. In general, the barrier will reduce the open-circuit voltage and impede efficient hole extraction. Although the barrier in CuSCN-D devices is small, and tunneling may overcome the barrier, the device performance with thick CuSCN-D (as thick as 750 nm) contradicts with this assumption. Therefore, we infer that holes should be transported through a transport energy level.

A close analysis of the density of states near the valence band edge reveals the existence of band-tail states, as shown in the shaded region of Fig. 3c. The possible contribution of carbon layer to the tail intensity has been ruled out by employing argon plasma etching before data acquiring. It is generally considered that these states within the bandgap originate from internal strain, composition variations, surface crystal or chemical defects^14^. In the case of solution-processed CuSCN films, acceptor-like states would appear near the Fermi level, due to structural defects or composition variations, through electrical field-effect measurements^42,44^. Previous reports of CuSCN-based dye-sensitized cells and field-effect transistors, along with DFT calculations, have predicted that the enhanced hole conductivity of CuSCN is due to acceptor-like states above VBM and they should occur near the Fermi level, which can even extend to the Fermi level owing to their broad energetic distribution^35,41,42^. The noticeable tail signals arranging from 0.37 eV to −0.25 eV observed in the valence band spectra clearly prove the assumption. By comparing the acceptor levels with experimental evidence, these defects are mostly suggested to be Cu vacancies. In addition to that, S vacancies (or equivalently, CN vacancies) are also possible to contribute to shallow degenerated levels, provided the vacancy occurs at a significant concentration ^41^.

Based on the above information and discussion, we constructed band diagrams to illustrate the hole conducting mechanism in Fig. 3d. Though CuSCN-D and Cu_2_O own close valence band edges, the conduction band minimum of CuSCN is much higher than that of Cu_2_O, resulting in a 2 eV bandgap difference. The large conduction band offset at the CuSCN\Cu_2_O interface generates a huge barrier for electrons to inject, thus electron-hole recombination is reduced in CuSCN. Although the valence band offset could hinder hole transporting from Cu_2_O to CuSCN, the band-tail states existence renders a smooth hole transport without experiencing the barrier (via valence band). Specifically, the hole injection barrier was reduced by this transport pathway, without heavy doping to shift Fermi level.

### The standalone device for overall water splitting

**Fig. 4:**
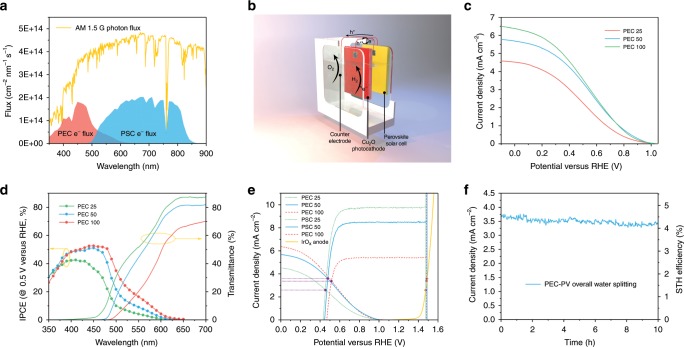
Standalone solar water splitting. **a** Plot of photon flux based on AM 1.5 G spectrum and expected electron fluxes of photocathode and PV calculated from wavelength-dependent IPCE responses of photocathode at 0.5 V versus RHE and PV at short circuit. **b** Illustration of PEC-PV tandem configuration based on CuSCN-incorporated Cu_2_O photocathode, PSC and IrO_x_ anode. **c** *J–E* responses of transparent Cu_2_O photocathodes with various thicknesses. Numbers in sample names denote the electrodeposition duration of Cu_2_O in minutes. **d** Wavelength-dependent IPCE of transparent Cu_2_O photocathodes biased at 0.5 V versus RHE and corresponding transmittance spectra. **e** Estimated tandem operating current density by overlaying *J–E* curves of photocathodes, *J–V* curves of PV cells and linear-sweep voltammetry of IrO_x_ anode. **f** Chronoamperometry of assembled PEC-PV tandem tested under simulated one-sun illumination in pH 5 buffered electrolyte with zero bias.

Though Cu_2_O photocathodes have experienced significant development in recent years, its photovoltage is still below the required 1.5 V for standalone overall water splitting^5,10–12^. Nevertheless, the solar-blind CuSCN layer is ideal for allowing the transmission of unabsorbed solar energy to a rear photoabsorber that could provide extra bias. Here, we choose the perovskite solar cell (PSC) as the rear absorber and simulated their individual photocurrent. Based on an AM 1.5 G photon flux spectrum and IPCE data of Cu_2_O photocathodes (front absorber) and PSCs (rear absorber, masked with the photocathodes), the electron fluxes are calculated as shown in Fig. 4a. With high IPCE, Cu_2_O is efficiently harvesting short-wavelength solar energy, yet leaving large amount of energy untapped due to its relatively large bandgap. As the rear photoabsorber, PSC collected solar photons transmitted by the Cu_2_O up to 850 nm. With two absorbers, the visible part of the solar spectrum is well-covered. Based on this tandem concept, we designed the arrangement of all parts in Fig. 4b, where the two basic parts of PEC photoelectrode and PSC cell are wired and placed closely back-to-back to minimize light scattering losses. The carrier paths are shown as well. When the system is illuminated, photo-generated electrons in Cu_2_O are collected and injected to the surface RuO_x_ catalyst to reduce water, while the holes will recombine with electrons from the PSC through the wire. Holes from PSC are conducted to the counter electrode back in the PEC cell for oxygen evolution reaction. Iridium oxide was selected as the counter electrode material due to its excellent catalytic activity ^45^.

As two parts are connected in series, their photocurrents have to match. To this end, it is necessary to find the balance between sufficient light absorption in Cu_2_O for good PEC performance and necessary transparency that permits proper PSC operation. The gold layer is removed while keeping the CuSCN layer, and three thicknesses of Cu_2_O (PEC 25, PEC 50, and PEC 100, where the numbers denote the duration of deposition in minutes) are explored by changing the duration of electrodeposition^13^. As shown in Fig. 4c, all performance is lowered without the Au contact due to increased resistance. Though the sample with the thinnest Cu_2_O shows only 4.5 mA cm^−2^ at 0 V versus RHE, the sample of 50 min electrodeposition exhibits similar performance to the 100 min one. Corresponding transmittance and IPCE at 0.5 V versus RHE were measured (Fig. 4d). Compared to the opaque sample, IPCE of all three samples are showing a global decline of around 7%. Two phenomena are noteworthy. First, as the film gets thicker, both the absorption and IPCE gets more significant at longer wavelength. This correlates well with the decline of the extinction coefficient with increasing wavelength^32^. In other words, thick films are necessary to increase photon harvesting in the long-wavelength region. Second, though the photons with energy smaller than the bandgap will interact weakly with the semiconductor, the transmittance at long-wavelength region (>600 nm) decreases with film thickness. We attribute this to an increase of light scattering caused by the larger roughness in thicker films.

The PSC we employed here is a PSC which exhibits an open-circuit voltage (V_OC_) of 1.076 V, short-circuit current density of 25.06 mA cm^−2^ and fill factor of 75.3% (Supplementary Fig. 23 and Supplementary Table 1). The PV performance with transparent Cu_2_O photocathodes as masks is also shown in Supplementary Fig. 23. Because of the undesirable energy loss with thick Cu_2_O mask in the long-wavelength region, the PSC with PEC 100 mask is giving only 4.48% conversion efficiency. However, the PSC with PEC 50 mask is giving similar efficiency (7.22%) value to the one with PEC 25 mask (8.17%). Corresponding IPCEs are shown in Supplementary Fig. 24. Clearly, considerable number of lower-energy photons that were wasted by Cu_2_O were not used by the PSC. Combining with the photocathode optical data, we suggest that optical effects at interfaces contribute significantly to photocurrent losses.

To predict the tandem operating current density, we overlapped the *J–V* curves of photocathodes, PSCs and the counter electrode (Fig. 4e). As both photocathodes and the counter electrode curves are fixed, the PV curves are shifted while matching the current densities of both crossing points with the other electrodes. Current densities of 2.59, 3.62, and 3.37 mA cm^−2^ are found for tandem systems with PEC 25, PEC 50, and PEC 100 front electrodes, respectively. Then PEC 50 was selected and connected with other parts as the schematic shown in Fig. 4b and tested with simulated 1 sun illumination for 10 h under two-electrode configuration. The current density is shown in Fig. 4f. A slightly larger current density of 3.72 mA cm^−2^ is observed at the beginning of the test compared to the estimated current density, which is probably due to activation in both the photocathode and the PSC^10,12,13^. STH efficiency, *η*_STH_, was calculated with by the following equation considering 100% Faradaic efficiency,

6 $$\begin{array}{*{20}{c}} {\eta _{{\mathrm{STH}}} = \frac{{j_{ph} \times 1.23V}}{{P_{in}}}} \end{array}$$

Where *j*_*ph*_ is the photocurrent density of the system, 1.23 V is the electromotive force for water splitting and *P*_in_ is the power of incident illumination, taken as 100 mW cm^−2^ for AM 1.5 G spectrum with 1 sun intensity. The corresponding STH efficiency was determined to be 4.55% and dropped to 4.15% (relative 8.8% loss) after 10-hour operation. As the photocathodes have been proved stable for tens of hours with gas quantification, we assume the degradation originates from the PSC due to high humidity around the PEC cell.

## Discussion

The value here represents one of the highest efficiencies achieved by unbiased solar water splitting devices using oxide photoelectrodes^46,47^. Nevertheless, we concluded several approaches to improve the tandem performance. First, considerable amount of energy was unexploited in the form of optical losses. Scattering and reflection at interfaces could be minimized by making a monolithic device, which integrate photoabsorbers and electrocatalysts into one neat piece. Quite a few high-efficiency devices fabricated in this style hold the potential of even lower cost because of simple structure^48,49^. Then, even though we applied electrocatalysts that are among the best performers, the slow kinetics for hydrogen evolution reaction (HER) and oxygen evolution reaction (OER) in near-neutral electrolyte leads to considerable burden on the system. To relieve the situation, one can either improve the catalysts by nano-structuring or transfer the system to more alkaline solution as most good OER catalysts work better in alkaline electrolyte. In the latter case, new protecting strategies should be developed to allow meaningful operation duration. Lastly, there are still space for both Cu_2_O photocathodes and PSCs to be improved further for this application. A careful examination of the Fig. 4e, reveals that the working potential does not lie at the maximum power point of either cells. To bring the curves together, higher photovoltage is desired. A photovoltage of 1 V is certainly not the limit of Cu_2_O considering its 2-eV bandgap. Since the photovoltage is determined by the Fermi level difference of its built-in junction, straight forward option is to shift the Fermi level of Ga_2_O_3_ by n-doping it with Sn^4+^, for example^12^. Moreover, as one of the advantages of PSCs, their composition is tunable and consequently their bandgap energies, indicating that large photocurrent densities can be sacrificed for higher V_OC_^50,51^.

PEC water splitting relies on efficient charge separation and transport. In this work, we have demonstrated, for the first time, the use of CuSCN as an effective HTL for Cu_2_O photocathodes, which resulted in overall enhanced performance. Two types of CuSCN films with different structures were fabricated by simple solution processes. Detailed analysis of optical, electrochemical and electronic characterization suggest that defective structure could be beneficial for hole conduction in CuSCN. Moreover, we discovered that hole transport between Cu_2_O and CuSCN was assisted band-tail states. At the end, to demonstrate the advantage of using CuSCN as HTL in Cu_2_O photocathodes, a standalone PEC-PV tandem was constructed delivering an STH efficiency of 4.55%. This efficiency stands as the highest among all Cu_2_O based dual-absorber tandems. Our results open up a promising path for future development of Cu_2_O-based solar water splitting systems.

## Methods

### Preparation of CuSCN films

Prior to deposition, FTO substrates were cleaned with ultrasonic in 2% Hellmanex solution (20 min), acetone (20 min), ethanol (20 min), and deionized water (20 min). Opaque samples are prepared on gold coated substrates while transparent samples are fabricated directly on cleaned FTO. 100 nm of Au was sputtered using Alliance Concept DP 650 with uniform mode. CuSCN-E is prepared by electrodeposition in aqueous solution containing 12 mM CuSO_4_ and equivalent amount of EDTA and KSCN whereas CuSCN-D is fabricated in aqueous solution containing 15 mM CuSO_4_, 67.5 mM DEA, and 45 mM KSCN. EDTA and DEA were added before adding KSCN to prevent Cu(SCN)_2_ precipitate formation. The pH of EDTA-contained precursor solution and DEA-contained precursor solution are 1.6 and 8.2, respectively. A standard three-electrode configuration was used for electrochemical deposition with Pt counter electrode and Ag/AgCl/sat. KCl reference electrode. All films were prepared using chronoamperometry technique with various duration. The deposition potentials of CuSCN-E and CuSCN-D respectively are −0.3 V and −0.45 V versus reference electrode.

### Fabrication of Cu_2_O photocathodes

all Cu_2_O photocathodes were prepared on CuSCN films for different conditions except conventional control samples with previously described method^12^. Cu_2_O was electrodeposited in a buffered copper sulfate solution. To prepare the solution, 7.98 g CuSO_4_, 67.5 g lactic acid, and 21.77 g K_2_SO_4_ were dissolved in 250 ml H_2_O followed by a pH adjustment to pH 10 with 2 M KOH aqueous solution. The electrodeposition of Cu_2_O was performed in galvanostat mode with current density of −0.1 mA cm^−2^. A large piece of platinum was used as the counter electrode and an Ag/AgCl/sat. KCl electrode is used as reference. Deposition duration was varied as specified in the main text. ALD was carried out for depositing Ga_2_O_3_ and TiO_2_ layers with Savannah 100 (Cambridge NanoTech) thermal ALD system. The thicknesses of Ga_2_O_3_ and TiO_2_ layers typically are both 20 nm and controlled by number of cycles of ALD. Gallium oxide was deposited using bis(μ-dimethylamino)tetrakis(dimethylamino)digallium (98%, Stream Chemicals) and TiO_2_ was deposited using tetrakis(dimethylamino)titanium (99.999%, Sigma). The chamber is stabilized at 150 °C and flushed with 10 sccm nitrogen gas (99.9995%, Carbagas). Specially, samples for 60-hour stability test were protected with 100 nm TiO_2_. RuO_x_ catalyst was photoelectrochemically deposited under galvanostatic mode as previously described^8^. Briefly, the deposition was carried out in 1.3 mM KRuO_4_ precursor solution for 6 min with constant current density of −28 μA cm^−2^ under simulated light illumination. Platinum wire was used as counter electrode.

### Fabrication of perovskite solar cells

The perovskite active layer was deposited on a freshly-prepared FTO/c-TiO_2_/m-TiO_2_ substrate and a two-step spin-coating method was applied with addition of 200 μL chlorobenzene. The perovskite solution precursor was prepared by dissolving lead iodide, formamidinium iodide and methylammonium iodide, methylammonium iodide and cesium iodide in dimethylformamide (DMF) and ultra-dry dimethyl sulfoxide (DMSO) under mild heating at 70 °C. All processes were done in a nitrogen glovebox. To coat hole transporting layer, Spiro-OMeTAD was dissolved in chlorobenzene with dopants of lithium bistrifluorosulfonyl imide and 4-tert-butyl pyridine and spin-coated on the perovskite film. The gold electrode was thermally evaporated onto the surface of HTL with mask.

### IrO_x_ electrode preparation

The IrO_x_ anode was prepared according to literature^13^. Briefly, a piece of titanium foil (99.7%, Sigma–Aldrich) was etched in boiling 1 M oxalic acid solution for 1 h. Then 30 μL of 0.2 M H_2_IrCl_6_ solution was drop cast on the foil followed by 500 °C calcination in air for 10 min. The above steps are repeated three times, resulting in the deposition of 6.3 mg of IrO_x_ deposited on the surface.

### Materials characterization

The phase composition of the as-prepared films was analyzed by X-ray diffraction (XRD) on an Empyrean system with PIXcel-1D detector and Cu Kα radiation. Diffraction patterns were recorded between 2*θ* of 10° and 80° at a scan rate of 1 min^−1^ with a step width of 0.02°. SEM images were collected with a high-resolution scanning electron microscope (Zeiss Merlin) equipped with the in-lens detector. X-ray photoelectron spectroscopy (XPS) measurements were performed with PHI VersaProbe II scanning XPS microprobe using a monochromatic Al Kα X-ray of 24.8 W power with a beam size of 100 μm. The spherical capacitor analyzer was set at 45% take-off angle with respect to the sample surface. All peaks are calibrated using adventitious C 1s peak of 284.8 eV to correct charge shift of binding energies after deconvolution. Valence band spectra were acquired after argon plasma etching to avoid the adventitious carbon layer contribution. Curve fitting was performed using PHI Multipak software after Shirley background subtraction. SEM images are collected using a high-resolution scanning electron microscope (Zeiss Merlin) with in-lens dector.

### Photoelectrochemical analysis

All photoelectrochemical and electrochemical analysis was done using SP-300 potentiostat with EIS module (BioLogic Science Instruments). PEC performance was characterized in homemade PEEK cells with three-electrode configuration, where Cu_2_O photocathode is the working electrode, Pt wire is the counter electrode and Ag/AgCl/sat. KCl is the reference electrode. Including stability tests, PEC performance is measured in a pH 5 buffer solution, which contains 0.5 M Na_2_SO_4_, 0.1 M sodium phosphate with an LCS-100 solar simulator (class ABB, Newport, with air mass 1.5 G filter) providing illumination. The intensity was controlled by light path distance which is determined by measuring the short-circuit current of a calibrated silicon diode with KG 3 filter. Calibration was done across the range between 300–800 nm dependent on photoabsorber bandgap. All linear-sweep voltammetry uses scan rate of 10 mV s^−1^. The IPCE was measured by comparing wavelength-dependent photoresponse at certain potential of photoelectrodes and a silicon photodiode (FDS100-CAL, Thorlabs) under light from 300 W xenon lamp through a monochromator (TLS-300XU, Newport). At each wavelength stair, current 5 s after wavelength shift was recorded. Electrochemical impedance measurement was carried out for CuSCN and Cu_2_O in 1 M Na_2_SO_4_ solution. The space-charge capacitance was recorded at various applied potential. Potential values were transformed to the reversible hydrogen electrode scale using the following equation:

7 $$\begin{array}{*{20}{c}} {E_{{\mathrm{RHE}}} = E_{{\mathrm{Ag}}/{\mathrm{AgCl}}({\mathrm{KCl}}\,{\mathrm{sat}}.)} + 0.197V + 0.059V \times {\mathrm{pH}}} \end{array}$$

Surface roughness of CuSCN was estimated by comparing the double-layer capacitance of the electrode/electrolyte interface to that of the mercury/electrode interface^34^. measuring the double-layer capacitance was determined using cyclic voltammetry (CV) measurements. The non-Faradaic potential window was first identified from initial CV scans and cyclic voltammetry scans with different rates were applied within the non-Faradaic potential window. The non-Faradaic current was then correlated against the scan rate, with the normalized slope (current density vs. scan rate). The electrochemically active surface area (ECSA) was calculated by dividing C_dl_ of the electrode by that of liquid mercury electrode. Thereafter, the roughness factor is found by comparing the ECSA to apparent electrode are.

### Optical measurements

Optical data of films were acquired using a UV-vis-NIR spectrophotometer (Cary) in transmission mode. All films are tested on FTO substrates using the FTO transmittance curve as background. Corresponding absorption spectra were derived according to Kubelka-Munk theory. The optical bandgap of Cu_2_O was determined from linear extrapolation of Tauc plots assuming direct allowed transition (*n* = 2).

### Hole mobility characterization

Hole only devices with the structure of FTO/Au/CuSCN/MoO_3_/Ag were fabricated to determine hole mobility of CuSCN-E and CuSCN-D. To construct the hole only device with the function of blocking the electron injection under bias, firstly, a layer of Au (100 nm) was sputtered on top of FTO. Then, EDTA- and DEA- CuSCN with a thickness of 1.5 μm were electrodeposited by the method described above. The CuSCN thickness was obtained from a calibration curve between electrodeposition time and film thickness (examined by cross-sectional SEM images). After that, the substrates were transferred to a vacuum chamber. Ten nanometer of MoO_3_ and 100 nm Ag were evaporated sequentially under a pressure of 1 × 10^−6^ mbar through a shadow mask (area defined as 28 mm^2^). The current density-voltage characteristics of the devices were tested by Keithley 2400 source measure unit under dark condition. The averaged mobility data is acquired from three devices and calculated from the forward *J*–*V* scan.

### Gas quantification

The amount of hydrogen evolved from a homemade gas-tight PEC cell was quantified by a gas chromatograph (Thermo Scientific) equipped with a Shincarbon column (Restek) and pulse discharge detector (PDD, Restek). The quantification is based on the calibration against certified gas standards with known concentration (Carbagas).

### Photovoltaic performance tests

Solar cells are measured using 300 W xenon light source (Oriel) with Schott K113 filter (Praezisions Glas & optic GmbH). Light intensity was calibrated by silicon photodiode for each measurement to match the standard air mass 1.5 G solar spectrum. A Keithley 2400 was used to drive and record current-voltage scan with scan rate of 50 mV s^−1^. Cell mask area is a 0.16 cm^2^ square. IPCE was carried out using monochromator (Arkeo & Ariadne, Cicci reserch) equipped with a 300 W xenon lamp.

### Tandem testing

Active area of transparent PEC electrode with RuO_x_ catalyst was defined by opaque epoxy (Loctite Hysol 9461). Homemade IrO_x_ electrode was positioned at the side in the pH 5 buffer electrolyte. Perovskite was centered and put closely against the back quartz window of the cell. The connections of PEC-PV and PV-IrO_x_ anode are built with copper wire by ultrasonic soldering. Specifically, the electron collecting contact of PV cell was connected to the back contact of photocathode while the hole collecting contact was connect to the anode. The same solar simulator was used to the one in PEC analysis. Current was recorded using chronoamperometry at zero bias potential.

## Supplementary information

Supplementary Information

## Data Availability

The data sets generated and analyzed during the current study are available from the corresponding author upon reasonable request.
