# Supplementary material for: Cu2O photocathodes with band-tail states assisted hole transport for standalone solar water splitting
Source: Nat Commun. 2020 Jan 16;11:318. doi: 10.1038/s41467-019-13987-5 (PMC6965123; doi:10.1038/s41467-019-13987-5)
Supplement: Supplementary file 1 — Supplementary Information [file 41467_2019_13987_MOESM1_ESM.pdf]

Cu_2O Photocathodes with Band-tail States Assisted Hole Transport for Standalone Solar Water Splitting

Pan et al.

Supplementary Information

Cu_2O Photocathodes with Band-tail States Assisted Hole Transport for Standalone Solar Water Splitting

Linfeng Pan[1], Yuhang Liu[2], Liang Yao[3], Dan Ren[2], Kevin Sivula[3], Michael Grätzel[2] and Anders Hagfeldt[1*]

[1]Laboratory of Photomolecular Science, Institute of Chemical Sciences and Engineering, École Polytechnique Fédérale de Lausanne (EPFL), Lausanne, CH-1015 Switzerland

[2]Laboratory of Photonics and Interfaces, Institute of Chemical Sciences and Engineering, École Polytechnique Fédérale de Lausanne (EPFL), Lausanne, CH-1015 Switzerland

[3]Laboratory of Molecular Engineering of Optoelectronic Nanomaterials, Institute of Chemical Sciences and Engineering, École Polytechnique Fédérale de Lausanne (EPFL), Lausanne, CH-1015 Switzerland

[*]E-mail: anders.hagfeldt@epfl.ch

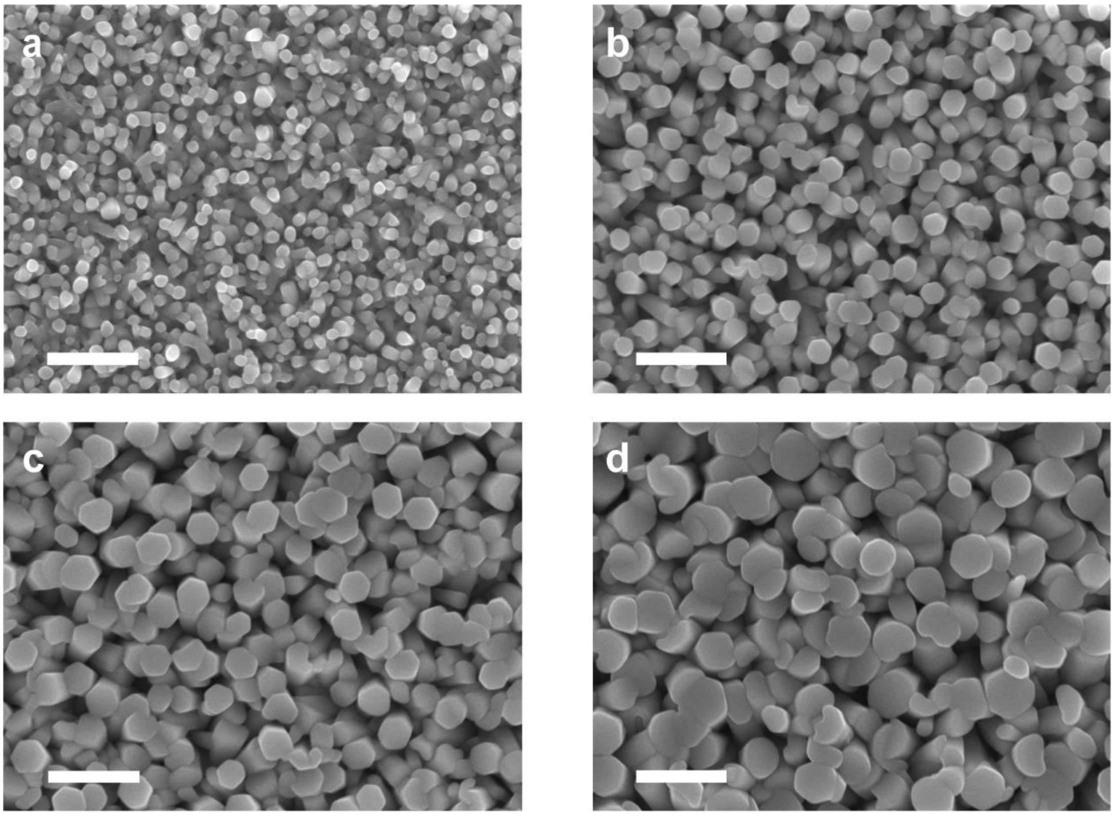

Supplementary Figure 1: Top-view scanning electron micrographs of CuSCN-E with **a,** 1 min, **b,** 4 min, **c,** 7 min and **d,** 10 min electrodeposition. Scale bar: 500 nm.

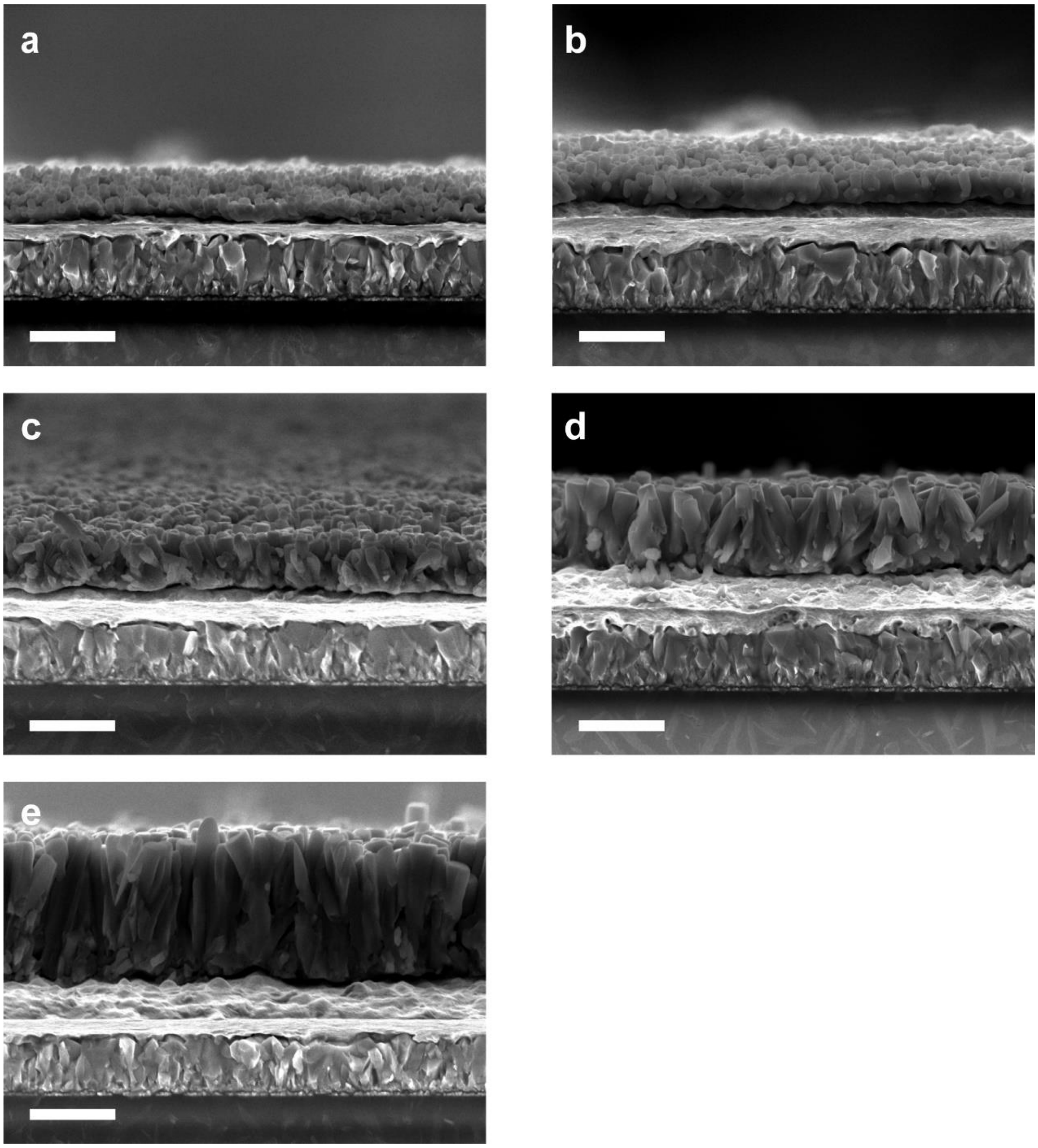

Supplementary Figure 2: Cross-section scanning electron micrographs of CuSCN-E with **a,** 1 min, **b,** 2 min, **c,** 4 min, **d,** 7 min and **e,** 10 min electrodeposition. Scale bar: 1 μm.

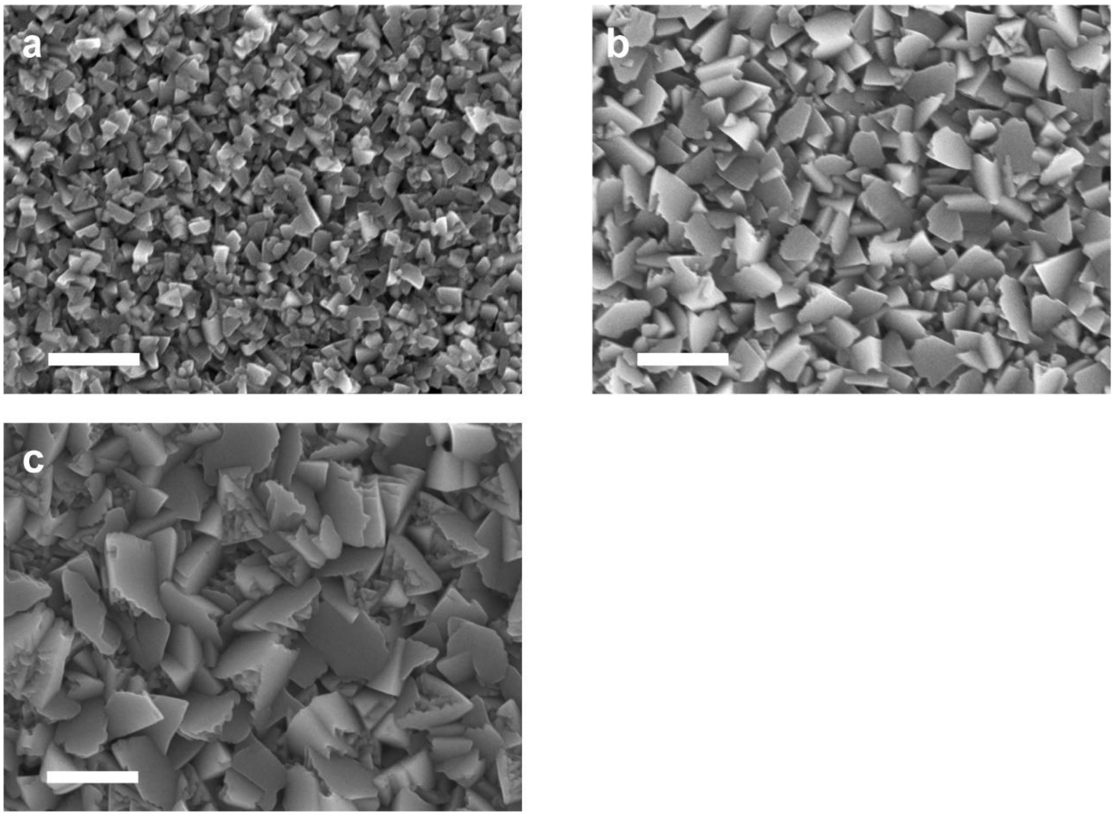

Supplementary Figure 3: Top-view scanning electron micrographs of CuSCN-D with **a,** 1 min, **b,** 5 min and **c,** 10 min electrodeposition. Scale bar: 500 nm.

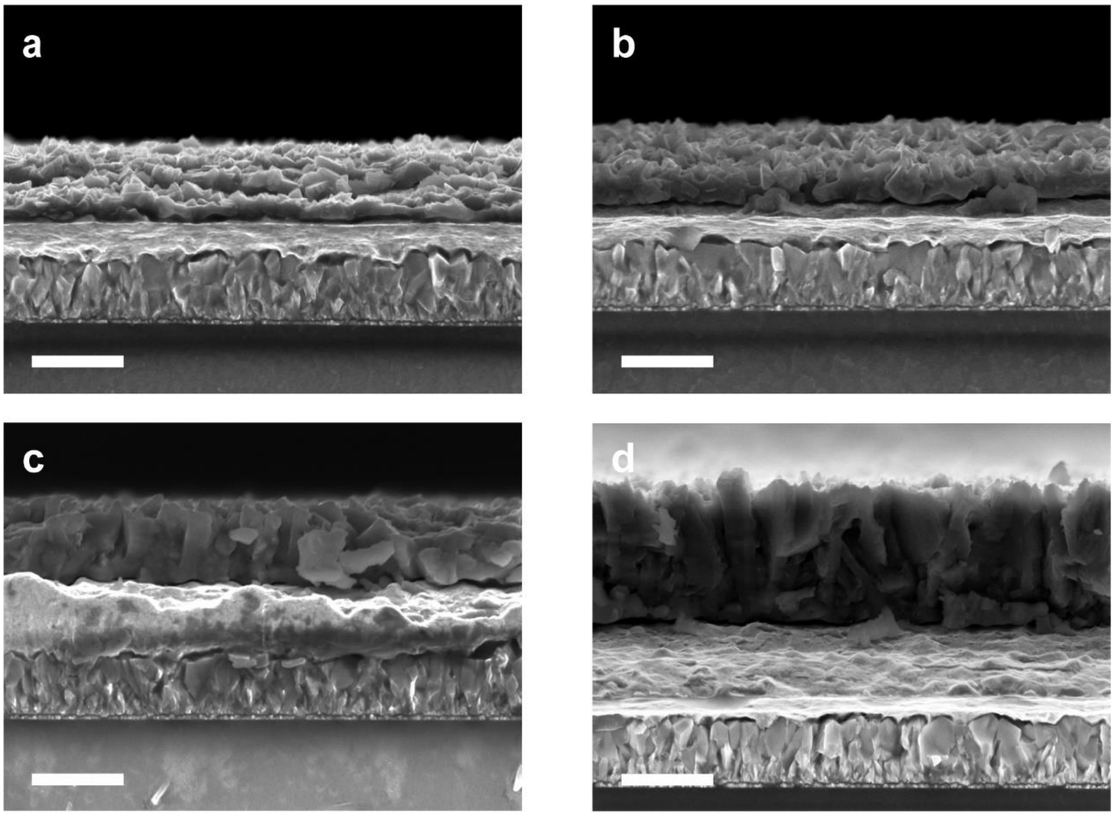

Supplementary Figure 4: Cross-section scanning electron micrographs of CuSCN-D with **a,** 1 min, **b,** 2 min, **c,** 5 min and **d,** 10 min electrodeposition. Scale bar: 1 μm.

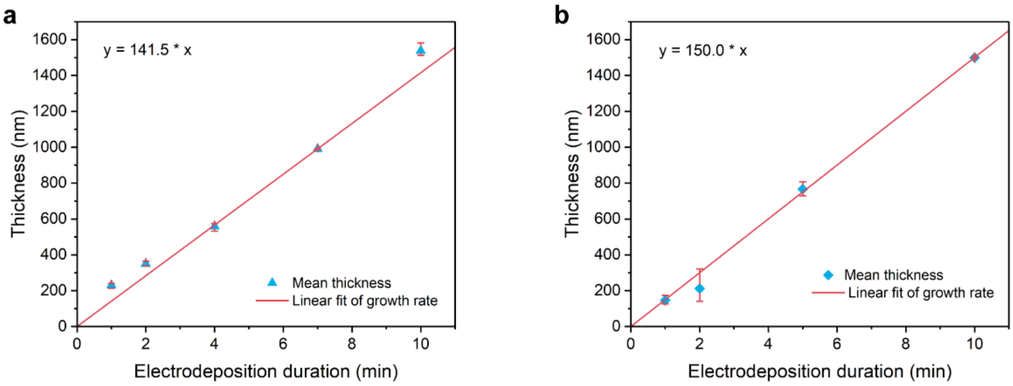

Supplementary Figure 5: CuSCN growth rate linear fitting. a, Growth rate of 141.5 nm per minute determined for CuSCN-E, **b,** Growth rate of 150.0 nm per minute determined for CuSCN-D.

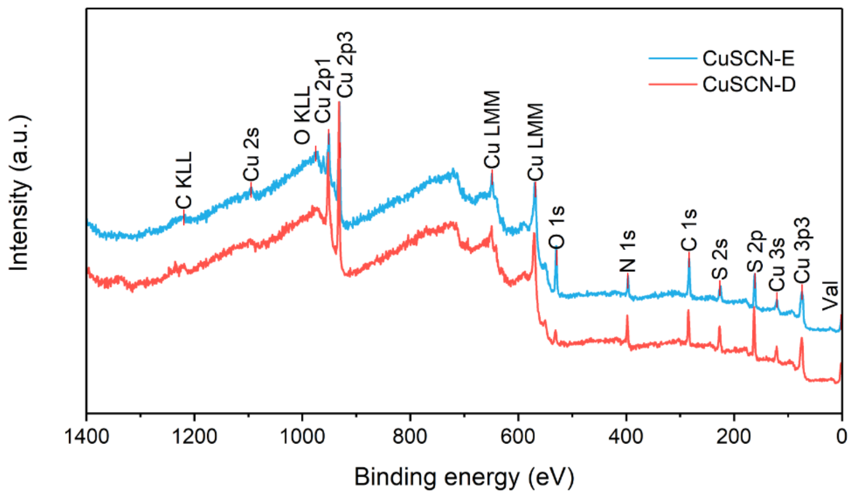

Supplementary Figure 6: XPS survey spectra of CuSCN films with 2 min electrodeposition.

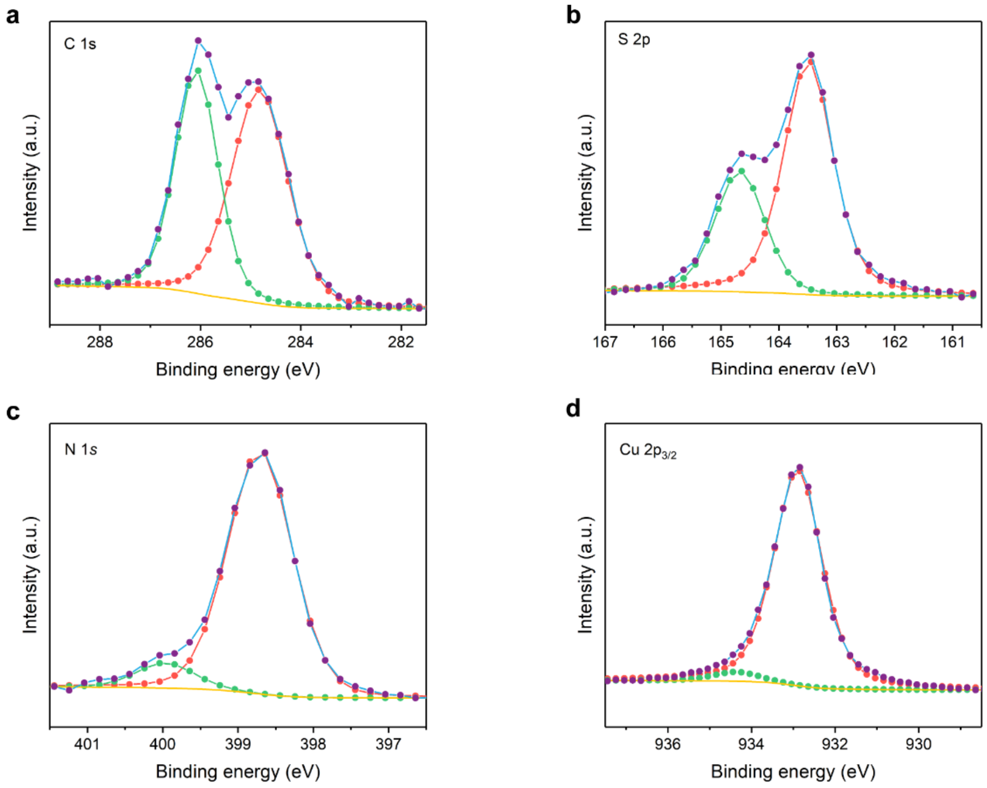

Supplementary Figure 7: High-resolution deconvoluted XPS spectra of CuSCN-E films with 2 min electrodeposition. **a,** carbon 1s spectra, **b,** S 2p spectra, **c,** N 1s spectra, **d,** Cu $2p_{3/2}$ spectra.

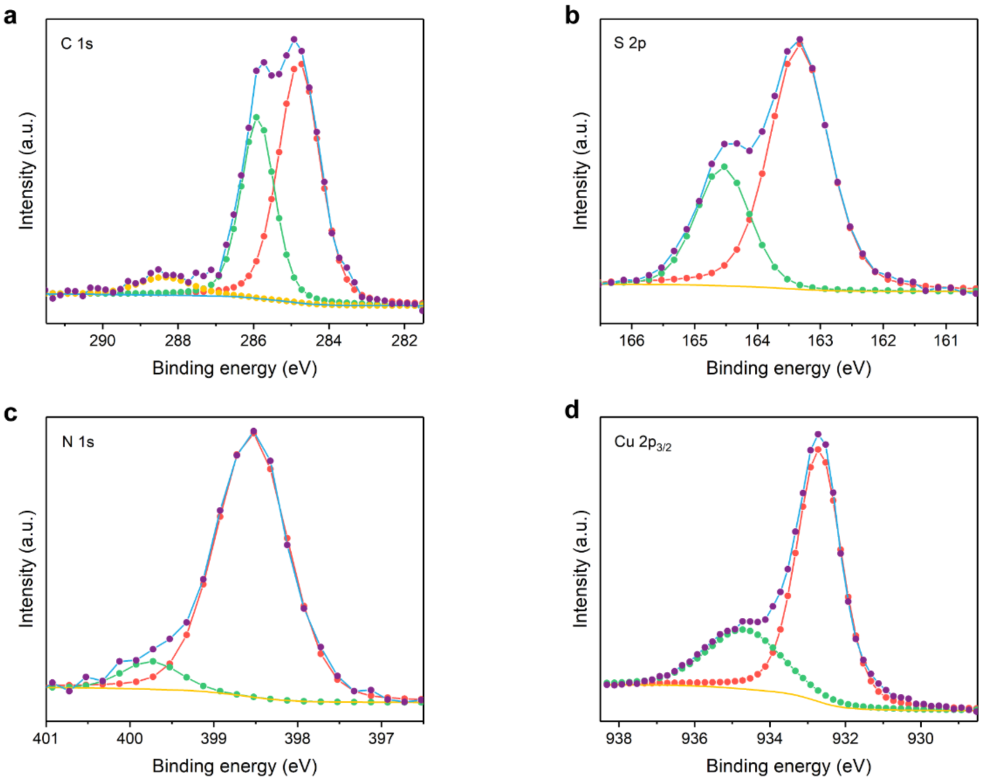

Supplementary Figure 8: High-resolution deconvoluted XPS spectra of CuSCN-D films with 2 min electrodeposition. **a,** carbon 1s spectra, **b,** S 2p spectra, **c,** N 1s spectra, **d,** Cu $2p_{3/2}$ spectra.

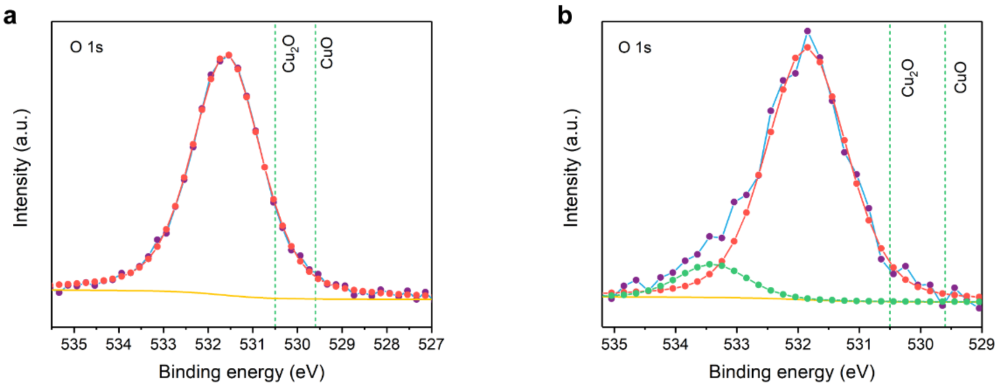

Supplementary Figure 9: High-resolution deconvoluted O 1s XPS spectra of **a,** the CuSCN-E film **b,** the CuSCN-D film.

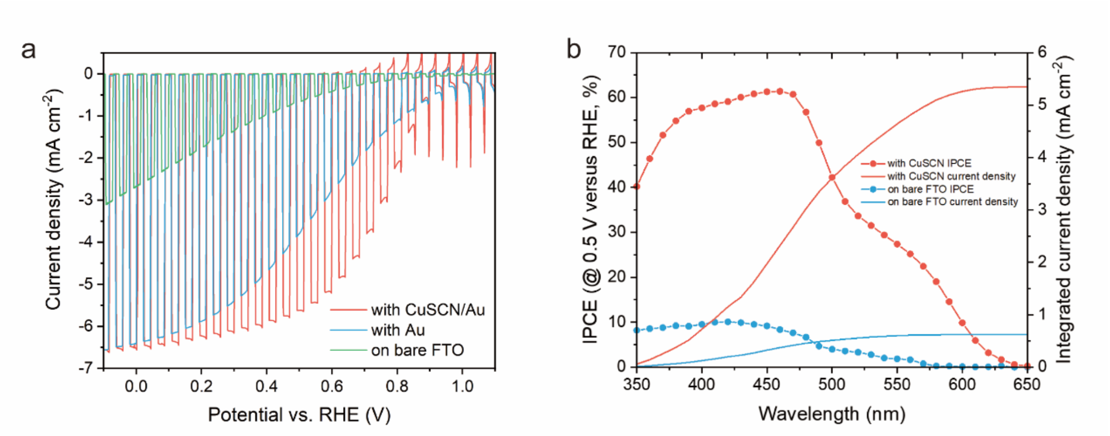

Supplementary Figure 10: Photoelectrochemical performance of Cu_2O photocathodes with **a,** Current density-potential (*J-V*) responses of Cu_2O photocathodes on various substrates. **b,** Wavelength-dependent IPCE and integrated current density of Cu_2O photocathodes.

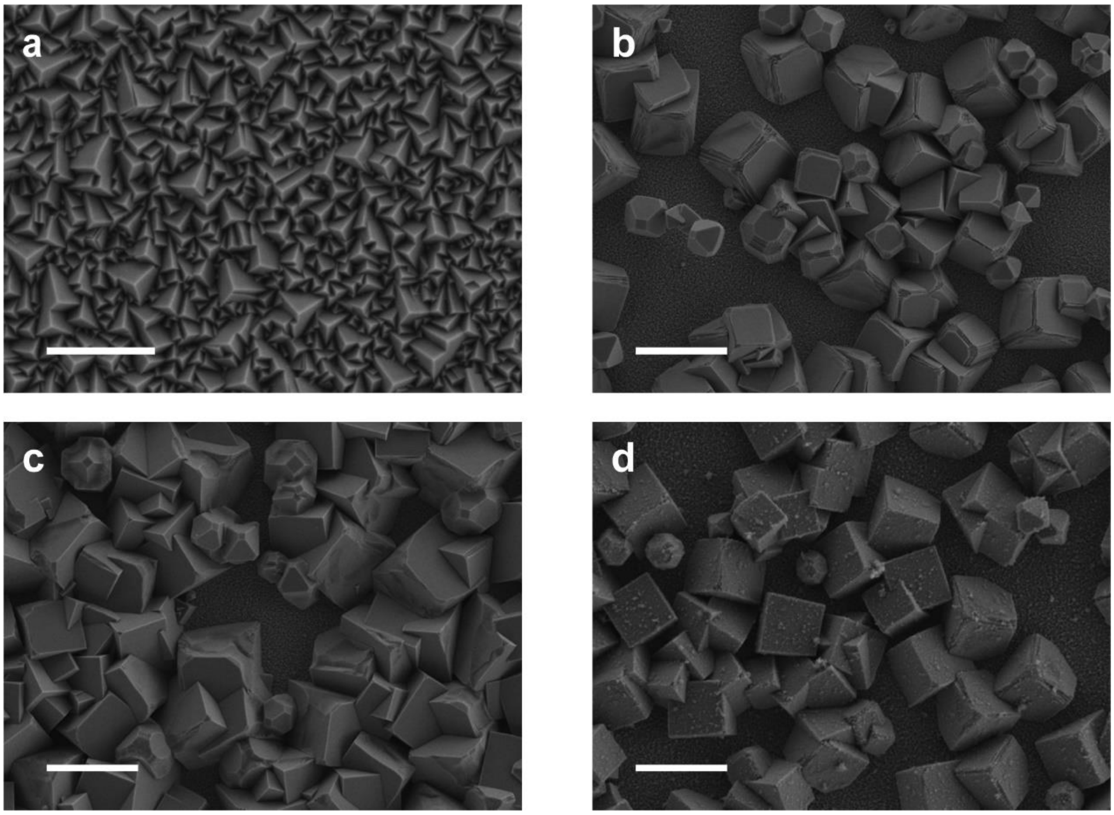

Supplementary Figure 11: Top-view scanning electron micrographs of Cu_2O photocathodes of **a,** the conventional Cu_2O photocathode, **b,** the Cu_2O photocathode on bare FTO, **c,** the Cu_2O photocathode with extended electrodeposition, **d,** Cu_2O photocathode on bare FTO after 40 hours of stability test. Scale bar: 2 μm in a and 5 μm for b, c and d.

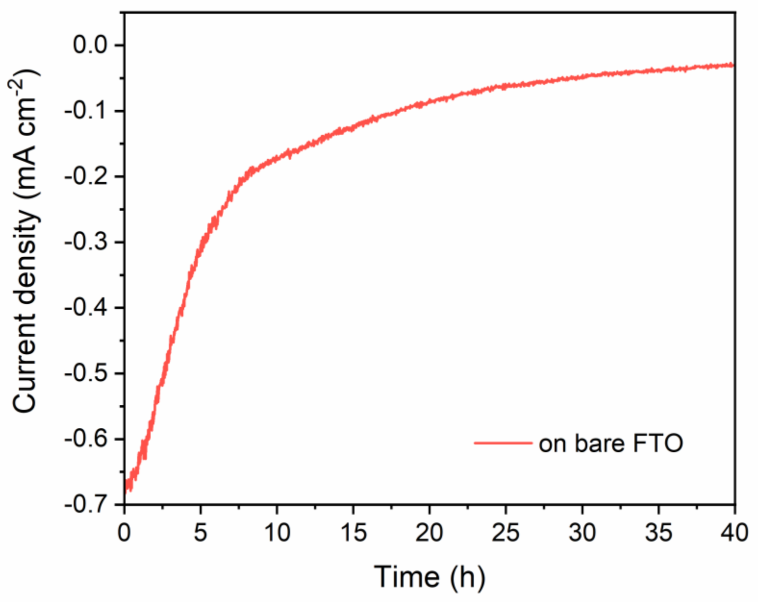

Supplementary Figure 12: Stability test of the Cu_2O photocathode with bare FTO substrates at fixed bias of 0.5 V versus RHE in pH 5 buffered electrolyte under simulated one-sun illumination.

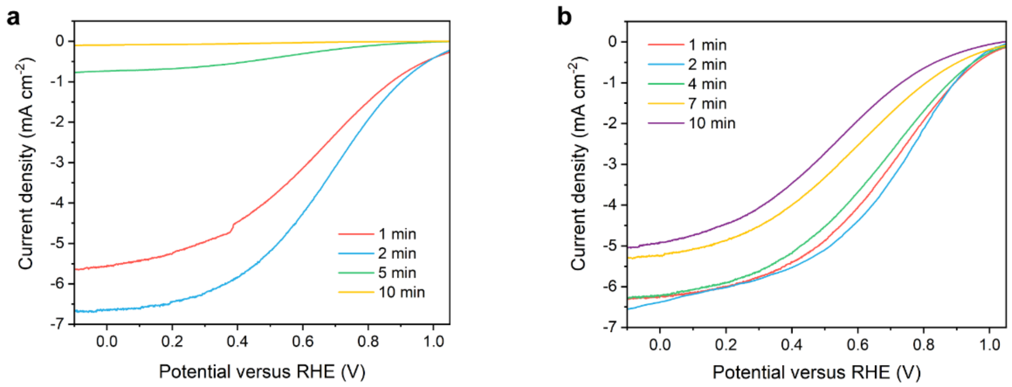

Supplementary Figure 13: Current density-potential (*J-V*) responses of Cu_2O photocathodes with **a,** CuSCN-E and **b**, CuSCN-D resulted from various electrodeposition duration as noted. All tests are carried out in pH 5 buffered electrolyte with simulated one-sun illumination. (AM 1.5 G spectrum, scan rate 10 mV/s, three-electrode configuration)

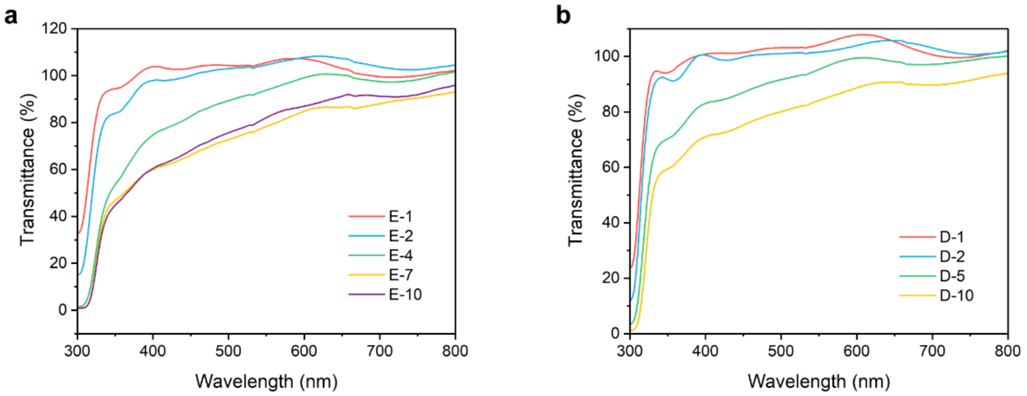

Supplementary Figure 14: Transmittance spectra of CuSCN measured on FTO glass. a, CuSCN-E samples with 5 thicknesses. **b,** CuSCN-D samples with 4 thicknesses. Numbers in sample names denote the electrodeposition duration in minutes.

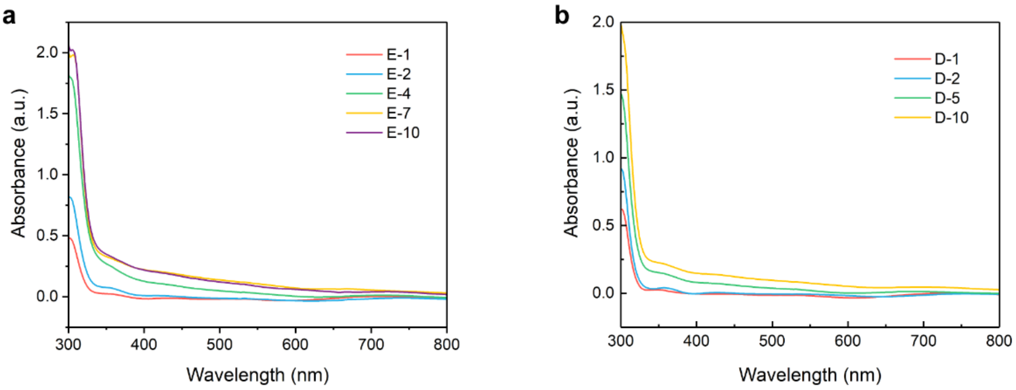

Supplementary Figure 15: Absorbance spectra converted from CuSCN transmittance data above. a, CuSCN-E samples with 5 thicknesses. **b,** CuSCN-D samples with 4 thicknesses. Numbers in sample names denote the electrodeposition duration in minutes.

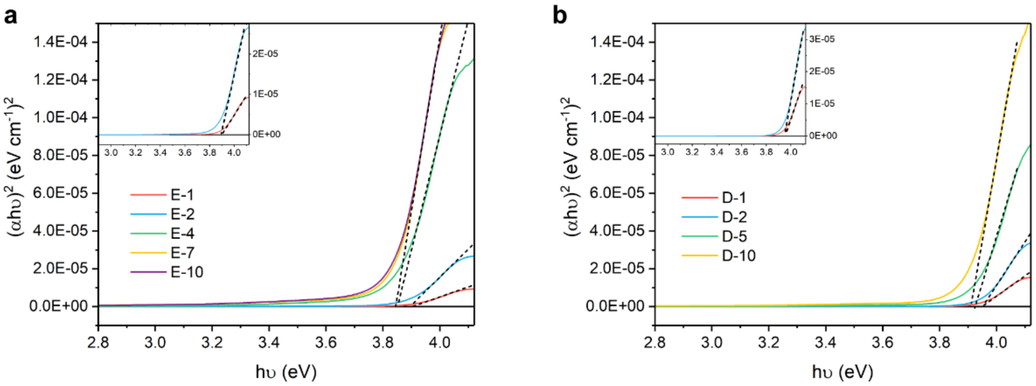

Supplementary Figure 16: Tauc plots of CuSCN on FTO substrates with linear extrapolation. a, CuSCN-E samples with 5 thicknesses. Inset graph shows enlarged extrapolation and cross of sample CuSCN-E-1 and CuSCN-E-2. **b,** CuSCN-D samples with 4 thicknesses. Numbers in sample names denote the electrodeposition duration in minutes. Inset graph shows enlarged extrapolation and cross of sample CuSCN-D-1 and CuSCN-D-2.

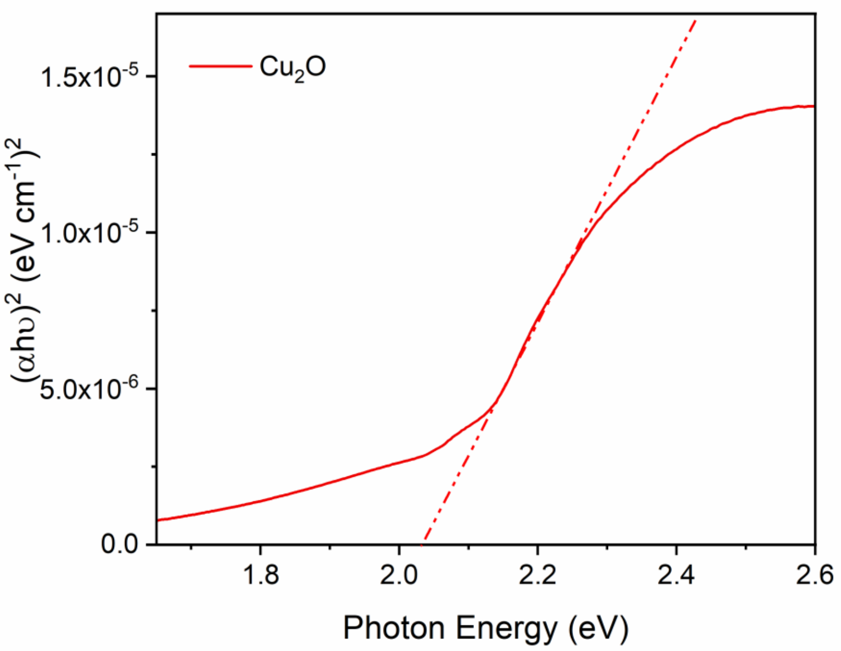

Supplementary Figure 17: Tauc plot of Cu_2O on FTO substrates with linear extrapolation.

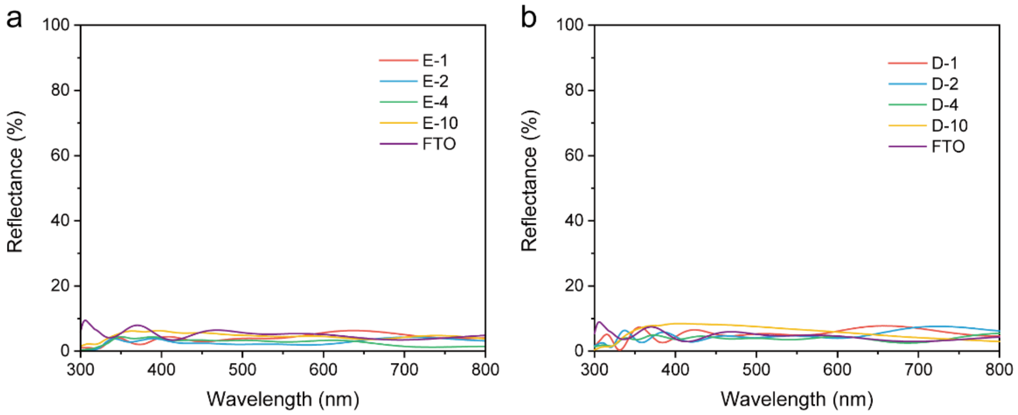

Supplementary Figure 18: Reflectance spectra of CuSCN measured on FTO glass and FTO black substrates. a. CuSCN-E samples with 4 thicknesses and FTO substrates. **b.** CuSCN-D samples with 4 thicknesses and FTO substrates. Numbers in sample names denote the electrodeposition duration in minutes.

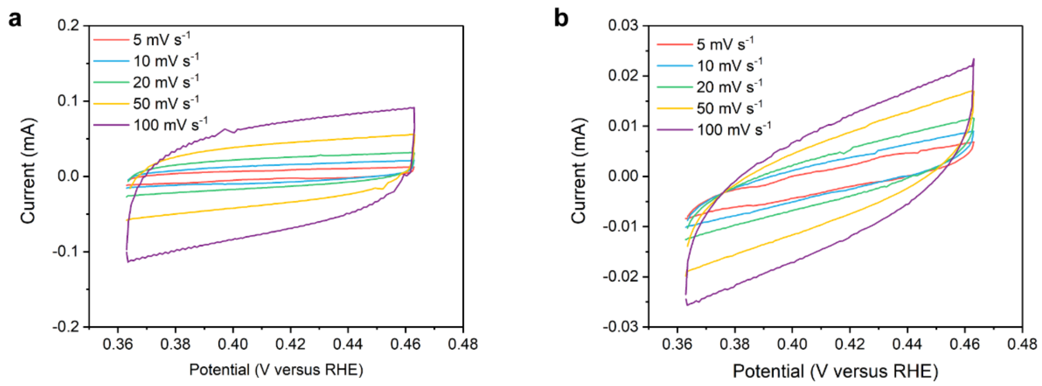

Supplementary Figure 19: Cyclic voltammograms of CuSCN films. Tests are carried out in 1 M Na_2SO_4 solution with various scan rates on **a,** CuSCN-E-2 and **b,** CuSCN-D-2.

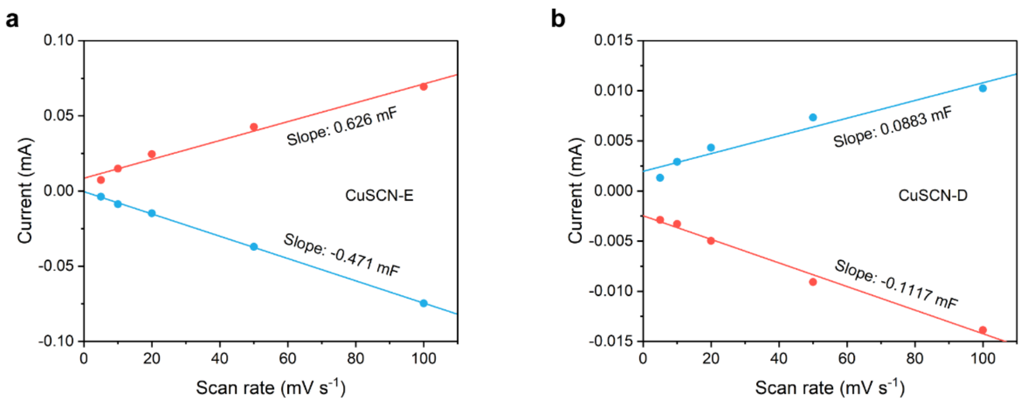

Supplementary Figure 20: The cathodic (blue) and anodic (red) charging currents as a function of scan rate with linear extrapolation. Currents are recorded at 0.41 V vsersus RHE.

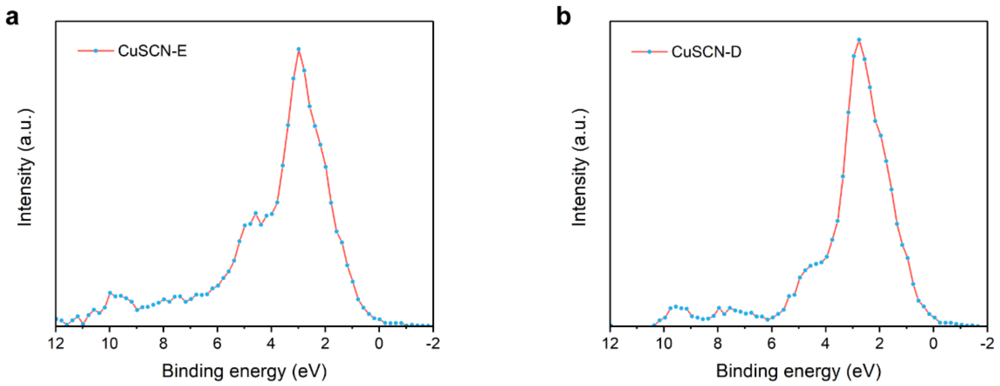

Supplementary Figure 21: XPS valence band spectra. a, Tested on sample CuSCN-E. **b,** Tested on sample CuSCN-D.

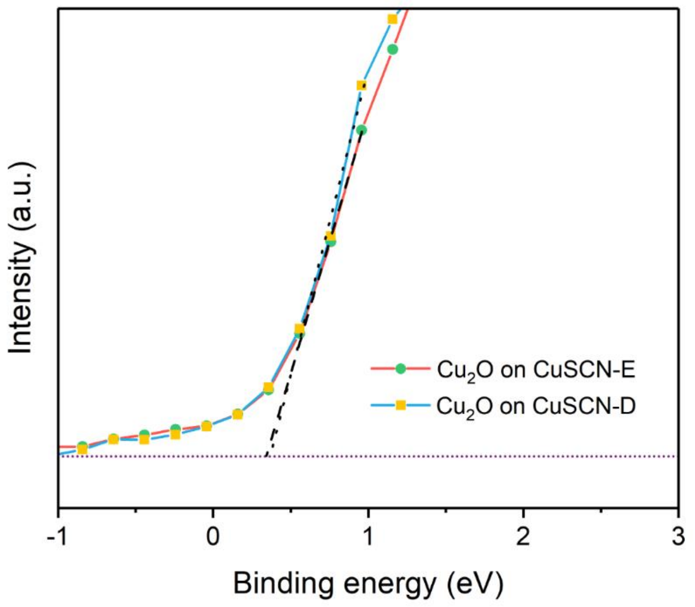

Supplementary Figure 22: Enlarged valence band spectra of Cu_2O.

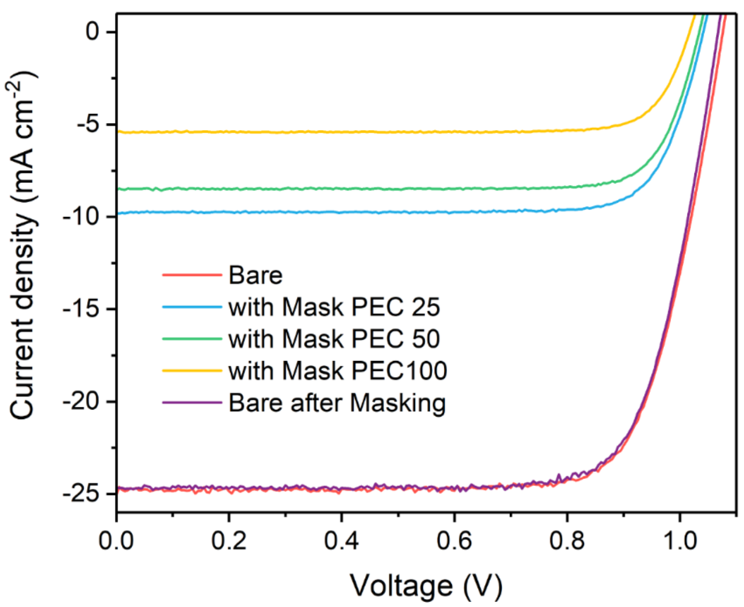

Supplementary Figure 23: The current density-voltage (*J-V*) curves of PSCs. Samples are tested under simulated AM 1.5 G illumination at 100 mW cm^{-2}.

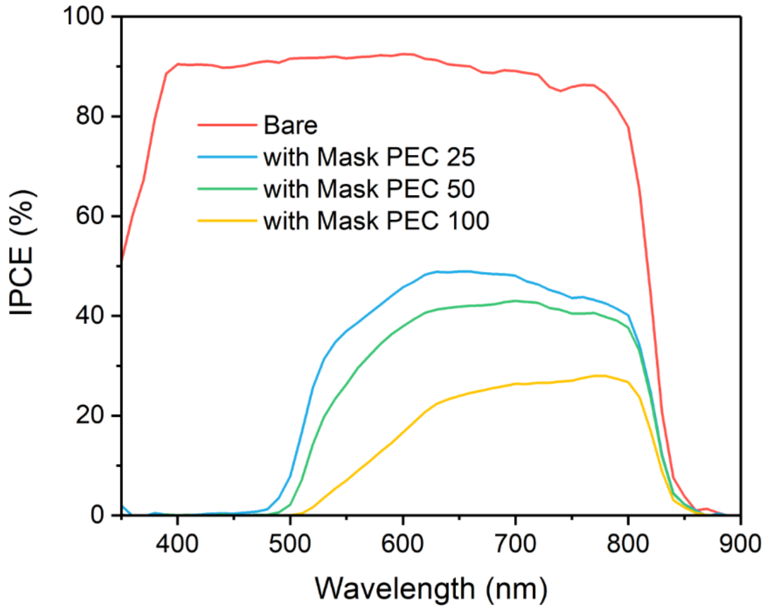

Supplementary Figure 24: Wavelength-dependent IPCE of PSCs.

Conditions	V_{OC}	J_{SC}	FF	PCE	J_{SC} (IPCE)
Bare	1.076	25.06	75.3	20.33	24.9
MASK PEC 25	1.041	9.81	79.8	8.17	9.34
MASK PEC 50	1.034	8.54	81.3	7.22	7.93
MASK PEC 100	1.017	5.43	81.4	4.48	4.38
Bare (after Masking)	1.068	24.68	76.6	20.2	N/A

Supplementary Table 1: Performance Summary of applied PSCs.

Supplementary discussion

PEC performance test of Cu_2O photocathodes on bare FTO substrates.

The sample with configuration of $RuO_x/TiO_2/Ga_2O_3/Cu_2O$/FTO was examined in the same electrolyte as other samples, Supplementary Figure 10. As the photovoltage of the photoelectrode is determined by the p-n junction, i.e. Cu_2O-Ga_2O_3 junction, all curves in the Figure a show photovoltage onset of aournd 1 V vs. RHE. However, the sample on bare FTO exhibits poor fill factor with current density of 2.9 mA cm^{-2} at 0 V vs. RHE. The deteriorated fill factor is often caused by increased impedance through the electric circuit. As the only modifed part is the substrates, we suggest that large number of photo-generated electrons and holes will recombine at the Cu_2O/FTO interface due to band energy mismatch. Whereas since gold has large work function of 5.1 eV,[1] it behaves as an ohmic contact to Cu_2O. Therefore, decent performance was achieved as previous studies. The wavelength-dependent IPCE was carried under the same condition as other Cu_2O photocathodes. No obvious change of absorption trend was found. The general reduction of IPCE along the spectrum suggests that the poor performance is due to either increased impedance or reduced light absorption. The latter was also comfimed by the following SEM images, Supplementary Figure 11. The conventional electrodeposited Cu_2O film shows continuous triangular crystal morphology in Supplementary Figure 11a while the Cu_2O deposited directly on FTO are cubic crystals, Figure Supplementary Figure 11b. More importantly, the crystals did not completely cover the FTO substrate, where large areas of substrates are exposed, Figure Xb. We then try to solve the problem by increasing the electrodeposition time to 200 min. Though the coverage was improved, substrates are still exposed, Supplementary Figure 11c. The broken film could be detrimental in two ways: first, the exposed area will not harvest light. Second, after ALD coating, the Ga_2O_3 layer could be in contact with the FTO substrate, causing considerable electron-hole recombination.

Stability test with the Cu_2O photocathode on bare FTO was also implemented as shown in Supplementary Figure 12. The same condition as applied in other stability test was employed. Starting from current density of 0.7 mA cm^{-2} at 0.5 V vs. RHE, the performance experienced a quick fall within 7 hours and a gradual decrease of current density in the next tens of hours. Accessing from the sample photos, Extra Figure 1, unlike the usual degradation which starts from weak spots of ALD protection layer, the FTO-based photocathodes shows a quick current density drop without severe damage of the photo-acitive layer, i.e. the scarlet Cu_2O layer. We suggest the performance decline could be resulted from degradation of Cu_2O which are in contact with FTO susbtrates, where photo-generated charges could oxidize and reduce the Cu_2O. Meanwhile, reported degradation pathway is also contributing to the overall performance decline as shown in Supplementary Figure 11d.[2] Though stability study is beyond the scope of this work, an ongoing study will cover degradation, one of the most important topics for photoelectrodes, in various conditions with different configuration.

Supplementary References

1. Son, M. K. *et al.* A copper nickel mixed oxide hole selective layer for Au-free transparent cuprous oxide photocathodes. *Energy Environ. Sci.* **10**, 912–918 (2017).
2. Paracchino, A., Laporte, V., Sivula, K., Grätzel, M. & Thimsen, E. Highly active oxide photocathode for photoelectrochemical water reduction. *Nat. Mater.* **10**, 456–461 (2011).
